# Recent advances towards azobenzene-based light-driven real-time information-transmitting materials

**DOI:** 10.3762/bjoc.8.113

**Published:** 2012-07-04

**Authors:** Jaume García-Amorós, Dolores Velasco

**Affiliations:** 1 Grup de Materials Orgànics, Institut de Nanociència i Nanotecnologia (IN2UB), Departament de Química Orgànica, Universitat de Barcelona, Martí i Franquès 1, E-08028, Barcelona, Spain

**Keywords:** kinetics, light-controlled materials, molecular switches, photochromic switches, push–pull azophenols, thermal isomerisation

## Abstract

Photochromic switches that are able to transmit information in a quick fashion have attracted a growing interest within materials science during the last few decades. Although very fast photochromic switching materials working within hundreds of nanoseconds based on other chromophores, such as spiropyranes, have been successfully achieved, reaching such fast relaxation times for azobenzene-based photochromic molecular switches is still a challenge. This review focuses on the most recent achievements on azobenzene-based light-driven real-time information-transmitting systems. Besides, the main relationships between the structural features of the azo-chromophore and the thermal *cis*-to-*trans* isomerisation, the kinetics and mechanism are also discussed as a key point for reaching azoderivatives endowed with fast thermal back-isomerisation kinetics.

## Introduction

**Figure 1:**
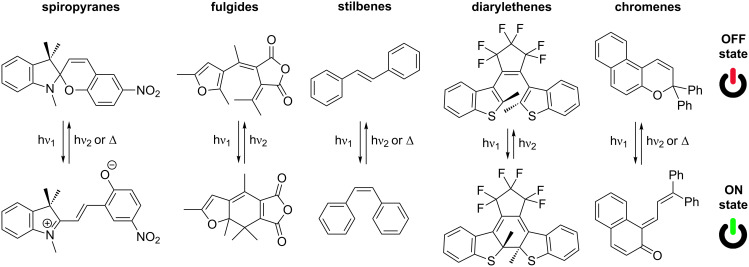
Some important families of photochromic compounds and their photochromic reactions.

Nowadays, there is an ever growing interest in molecular switching materials because of the very rapid development of modern technology. This tendency arises from the great applicability of such systems as active data elaboration, storage and communication elements in many devices, such as optical systems for opto-electronics, holographic materials and multicolour displays. Hence, there is a high motivation for the molecular design and study of innovative materials for this purpose. “Molecular switches” denote molecular systems, either small molecules or supramolecular species, that can be reversibly shifted between at least two different states [1]. As a consequence of the molecular change produced by the application of an external input energy, a significant modification of some of the properties of the molecule, e.g., mechanical, magnetic, electrical, optical, etc., is induced. Many diverse organic molecules have been investigated for molecular switching applications. Based on these, monomolecular photochromic [2–4], fluorescence [5–6], chirality [7–9], redox [10] and acid–base [11–13] switches have been successfully reported during the past few decades. The interconversion between the different states of the molecular switch can be performed by a great variety of environmental stimuli, which can be classified into three main groups: light energy, electrical energy and chemical energy (pH, solvent, the presence of a determined metal or ligand, etc.). Moreover, in some cases, a combination of some of them is required so as to endow the final material with multifunctionality. In comparison with chemical stimulation, both electrochemical and mainly photochemical excitation can be switched back and forward easily and rapidly. On the other hand, the use of light and electricity means that not only the switching of the probe but also the monitoring of the operation can be performed simultaneously. Furthermore, these two external input energies are the easiest to use in macroscopic device engineering. Specifically, light-responsive materials are themselves attractive because light allows a clean, quick and remote operation without the need for direct contact to the material. Photochromism is defined by IUPAC as a “light-induced reversible change of color” [14]. Organic photochromic molecular switches are based on molecules that can be reversibly interconverted, with at least one of the reactions being induced by light excitation, between two forms with different absorption spectra [15]. These two forms differ also in many other physical properties, such as their redox potential, fluorescent intensity, acid/base strength, dielectric constant, dipolar moment, molecular shape, and so on. Traditionally, this type of organic system has been widely used for analytical purposes, but at the present moment, they are widely required for many technical uses, for instance, binary systems for logic gates in computing or signal transmission devices. Many different organic photochromic molecules are known in photochemistry, such as azobenzenes, stilbenes, spiropyranes, fulgides, diarylethenes and chromenes among many others (Figure 1) [16]. The photochromic processes that take place when such compounds are illuminated can be divided in three different classes: *trans*–*cis*–*trans* isomerisations, photo-induced ring-closing reactions and photo-tautomerism [17].

Depending on the thermal stability of the photogenerated isomer, photochromic systems can be classified in two categories [18]:

● P-type (photochemically reversible type); these do not revert to the initial isomer even at elevated temperatures (e.g., fulgides and diarylethenes).

● T-type (thermally reversible type); the photogenerated isomer thermally reverts to the initial form (e.g., azobenzenes, stilbenes or spiropyranes).

**Figure 2:**
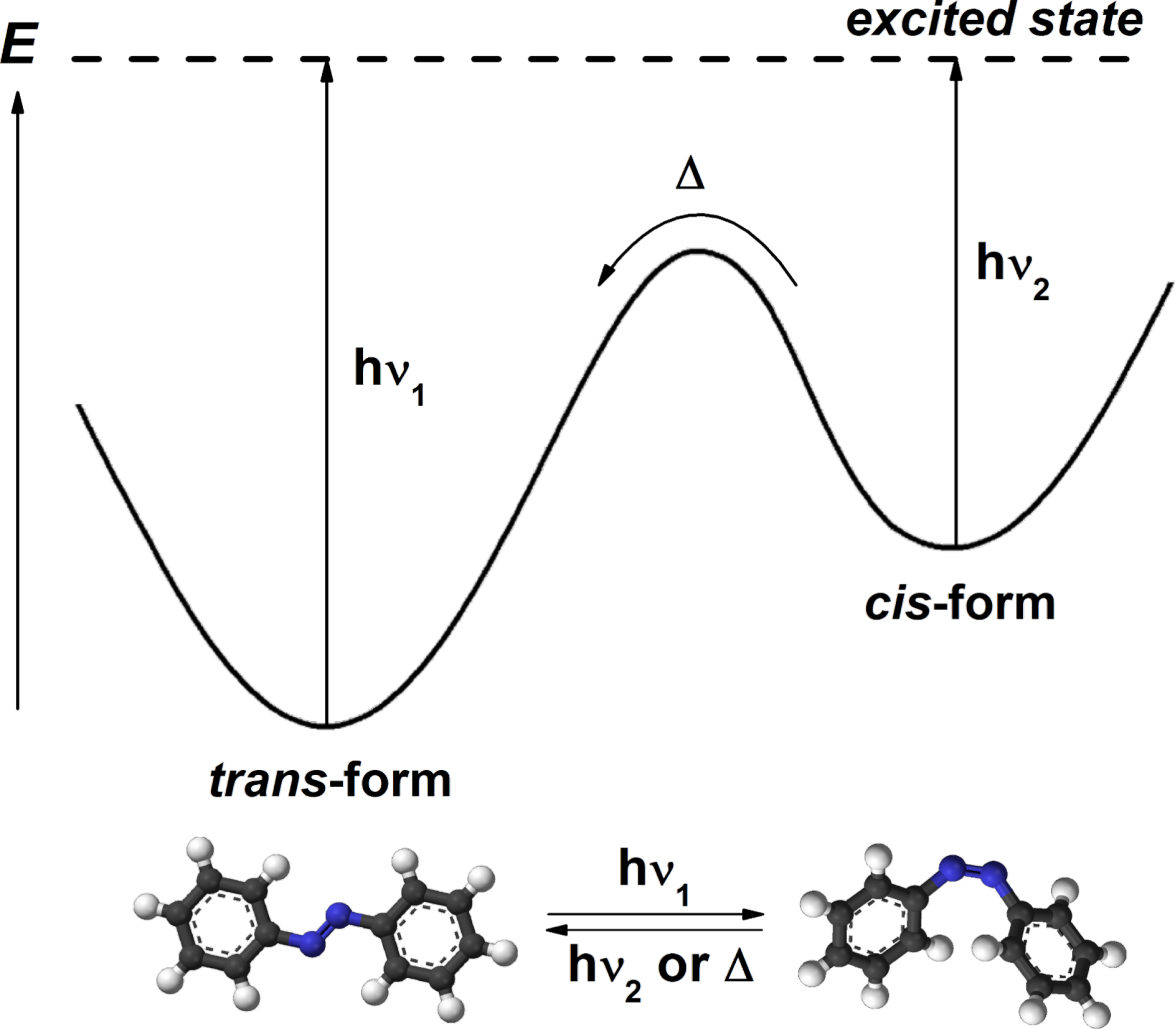
Photochromism of azobenzene derivatives and energetic profile for the switching process.

One of the most used organic chromophores for optical switching applications are certainly azobenzenes. Azobenzene, a photochromic T-type system, exhibits a reversible isomerisation process between its *trans* and *cis* isomers of different stability. In this process, the photoreaction simply causes the rearrangement of the electronic and nuclear structure of the molecule without any bond breaking. Moreover, the totally clean reverse *cis*-to-*trans* conversion also takes place thermally in the dark, spontaneously (Figure 2). It should be also noted that if the *cis*-to-*trans* back reaction is induced by visible-light excitation instead of thermally, then the formation of phenyl radicals may occur and dediazotation can be observed [19–20].

Azobenzene-based photochromic systems are under kinetic control; that is, after a photochemical conversion, whose rate depends mainly on the intensity of the excitation beam, the spontaneous thermal back reaction occurs. While the photo-induced *trans*-to-*cis* isomerisation reaction can be performed in a few femtoseconds with a light source that is powerful enough, the rate of the thermal *cis*-to-*trans* back reaction depends greatly on the chemical architecture of the system. It is well known that the appropriate modification of the substitution of the azobenzene core is one of the main factors that allows modulating the thermal relaxation rate of azo-dyes and, therefore, determines the response time of the photochromic molecular switch. The response time of the photochromic switch is a key feature in its overall performance. This parameter is directly related with the thermal isomerisation rate of the photo-sensitive azo-dye in the dark, that is, with the relaxation time of the *cis* isomer of the azo-moiety. Slow thermally back-isomerising azoderivatives are valuable photoactive basic materials for information storage (memory) purposes. A molecular-level memory should be stable and easy to write, and, moreover, its switched form should be stable but readily erasable when necessary. However, for azobenzene-based photochromic switches that can be used in real-time information-transmitting systems as well as optical oscillators, it is essential that the return to the thermodynamically stable *trans* form in the dark occurs as fast as possible, that is, as soon as the optical stimulation is removed the molecule should revert to its initial state. In fact, the information is expected to be transmitted at the molecular scale with response times ultimately within the nanosecond or picosecond range. Even though some chromophores, such as spiropyranes, have been already proved to show very fast thermal back reactions occurring within hundreds of nanoseconds [21–23], reaching such fast relaxation times for azobenzene-based photochromic molecular switches is still a challenge. This aim has attracted a great deal of attention over the past few years due to the potential application of fast photoactive azobenzene-based materials in micropumps and autonomous valves that simulate the beating of the heart; photoactive polymers that mimic cilia movement; and artiﬁcial muscles for robotics or molecular rotary motors, among others [24–33]. Moreover, besides photochromic switching, azobenzenes with different isomerisation rates have been successfully applied very recently for both photoelectronic [34–37] and photomagnetic actuating purposes [38].

**Figure 3:**
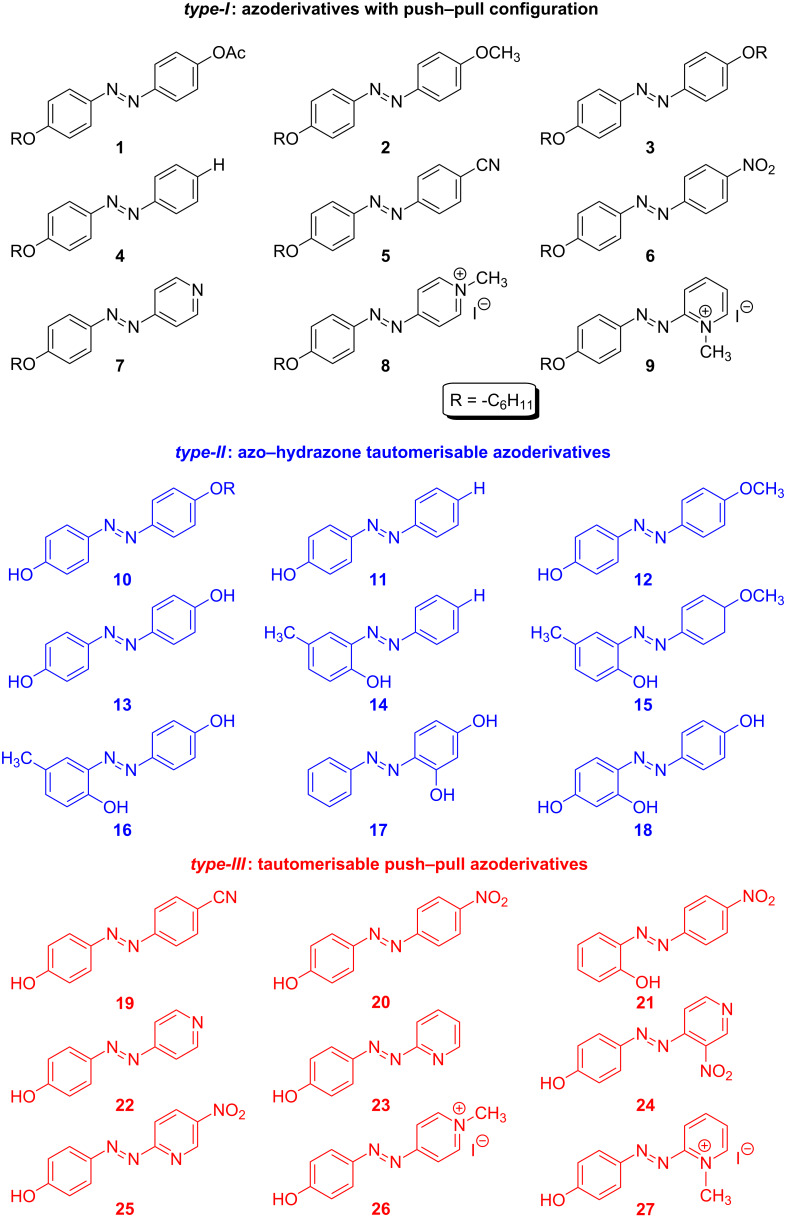
General overview of the different types of azoderivatives presented in this review.

In this review, we present the efforts made in our research group over the past few years regarding the molecular design of new photosensitive azoderivatives endowed with very fast *cis*-to-*trans* thermal isomerisation processes, with the main aim of transmitting optical information beyond the time scale of microseconds, as well as to produce molecular materials with high oscillation frequencies in their optical properties. In addition, the possible design of such materials in such a way that they would be soluble in water (an environmentally friendly solvent) could be a noteworthy benefit since it opens a new door for further uses of these molecules in both biological and medical applications, such as photochromic ion-channel blockers [39], or allowing the photocontrol of neurotransmitters in the central nervous system [40–41]. Additionally, some of the azobenzene-based photochromic switches reported heretofore require temperatures substantially above 298 K to achieve a rapid thermal isomerisation of the azo-dye [42–43]. This fact limits substantially the usefulness of the final device. In this way, finding new azoderivatives exhibiting fast isomerisation rates at room temperature is a challenging point of research and, consequently, also one of the main topics of the present work. With this purpose in mind, three different types of azoderivatives were designed and studied (Figure 3). Firstly, we investigated such azoderivatives that exhibit a 4-donor-4’-acceptor substitution (*type-I*), that is, bearing a push–pull configuration. Secondly, kinetic studies of different azophenolic systems (*type-II*) will be presented, since these azoderivatives exhibit a fast thermal back reaction due to their capability to establish an azo-hydrazone tautomeric equilibrium. Both *type-I* and *type-II* azoderivatives allowed the transmission of information within a few of milliseconds. Finally, those azo-dyes that combine both phenomena (*type-III*) were tested, registering action speeds down to some tens of microseconds.

## Review

### *Type-I* azoderivatives: photochromic switches acting within the millisecond time scale, based on chromophores with a push–pull configuration

**Figure 4:**
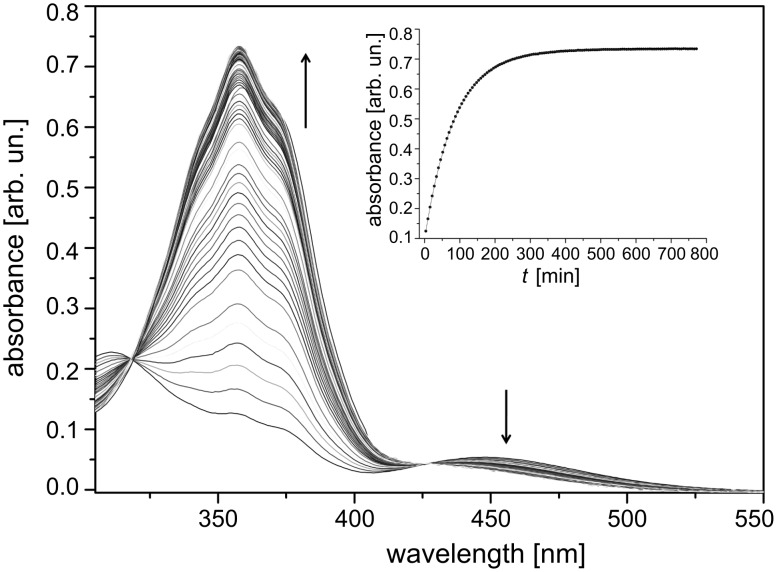
Changes in the electronic spectrum of a **3** *cis*-to-*trans* isomerising ethanol solution at 45 °C (Δ*t* = 360 s, [**3**] = 30 μM). From [44] – Reprinted with permission from *J. Phys. Chem. B.* © 2010, American Chemical Society.

The electronic spectra of the stable *trans* isomer of *type-I* azoderivatives **1**–**4**, display a high-intensity band peaking at ca. 355 nm, corresponding to the π→π^*^ transition, and a weak broad-band signal at ca. 450 nm, associated with the n→π^*^ transition. Upon UV-irradiation, the *trans*-to-*cis* photoisomerisation occurs, producing a decrease in the intensity of the 355 nm band and an increase in the 450 nm signal until the photostationary state is reached, that is, the inverse process of that shown in Figure 4 [44].

The kinetics of the thermal *cis*-to-*trans* isomerisation process for azo-dyes **1**–**6** were analysed by conventional UV–vis spectroscopy. Since these azoderivatives are not water-soluble, ethanol was used as a solvent instead of water. The unimolecular thermal *cis*-to-*trans* isomerisation process in the dark obeys Equation 1.

[1]
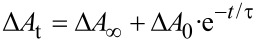


where Δ*A*_t_, Δ*A*_0_ and Δ*A*_∞_ correspond to the absorbance change at time *t*, *t* = zero and *t* = infinite, respectively, and τ is the relaxation time of the corresponding *cis* isomer. The relaxation times were derived from plots of the absorbance, Δ*A*, versus time by fitting Equation 1 to the experimental data, which perfectly described a first-order profile.

**Figure 5:**
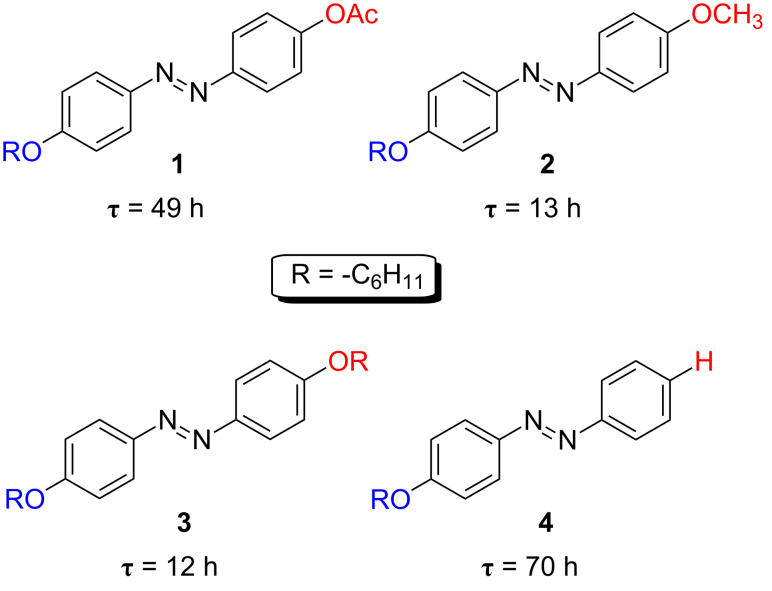
Chemical structure and thermal relaxation time in ethanol at 298 K, τ, for the slow thermally-isomerising *type-I* azoderivatives **1**–**4**.

Azocompounds **1**–**4** presented very slow thermal *cis*-to-*trans* isomerisation kinetics showing relaxation times for their thermal back reactions from several hours to a few days (Figure 5) [44].

**Figure 6:**
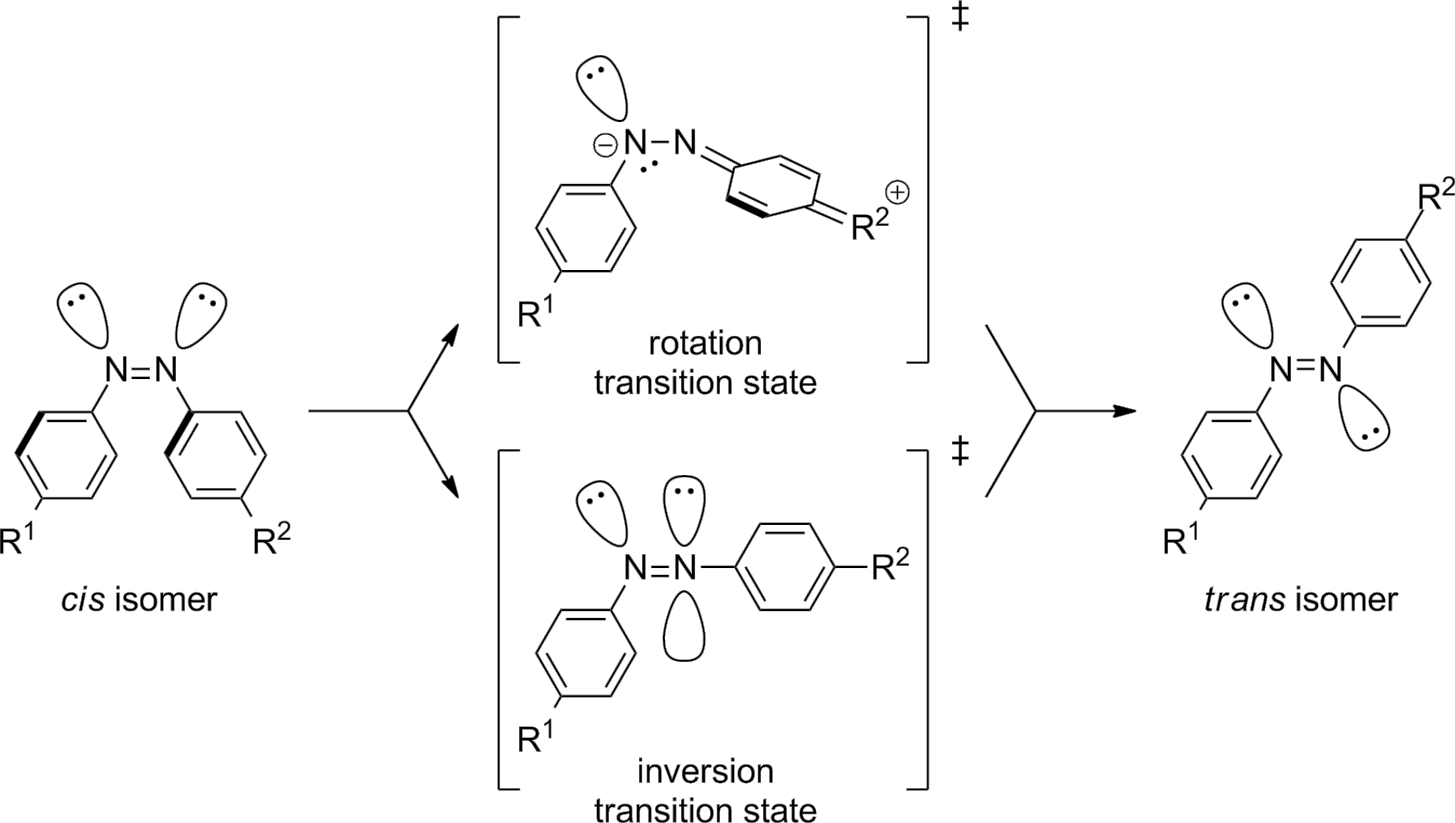
Rotation and inversion mechanisms proposed for the thermal *cis*-to-*trans* isomerisation processes of azobenzenes.

The rate of the thermal *cis*-to-*trans* back reaction is clearly determined by the intimate mechanism through which the process takes place. This mechanism has attracted much attention for many years and has given rise to controversy. For the process, two different extreme mechanisms have been proposed (Figure 6); one involving a simple rotation around the N–N bond [45–46], and another implying an inversion, in-plane lateral shift, through a linear transition state [47–49]. However, recent advances in both gas phase and in solution point out that some mixed mechanisms, such as the concerted inversion or the inversion-assisted rotation, can also be possible pathways for the *trans*-to-*cis* photoisomerisation of azobenzenes [50–52].

**Figure 7:**
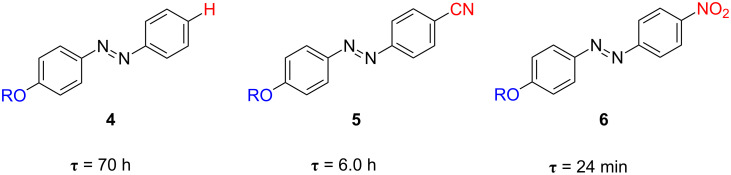
Effect of the presence of the electron-withdrawing cyano and nitro groups on the thermal relaxation time in ethanol at 298 K, τ, for the *type-I* azoderivatives **5** and **6**.

The measurement of the volumes of activation for these processes provides unequivocal evidences regarding the operation of the inversion or rotation isomerisation mechanism [53–54]. The volumes of activation for the thermal *cis*-to-*trans* isomerisation process of azobenzenes **1**–**3** were all close to zero, as expected [44]. These values perfectly correlate with those found in the literature for the azobenzene-bridged crown-ether 3,3’-[1,10-diaza-4,7,13,16-tetraoxa-18-crown-6]-biscarbonylazobenzene, which thermally isomerises by the inversion mechanism, because of its impossibility to evolve through the rotational pathway due to structural restrictions [55]. Therefore, not only acetylated (**1**) and alkoxylated (**2** and **3**) azo-dyes, but also the parent nonsubstituted one (**4**), thermally back-isomerise through the inversion mechanism [44]. Both 4-cyano-4’-(5-hexenyloxy)azobenzene (**5**) and 4-(5-hexenyloxy)-4’-nitroazobenzene (**6**) show a gradual increase in the strength of their push–pull electronic distribution as a consequence of the placement of the electron-withdrawing cyano or nitro groups in one of the *para*-positions of the azobenzene core. This structural variation produces a red shift of the π→π^*^ transition to 375 nm with respect to that of the parent nonsubstituted azo-dye **4** (355 nm). In azoderivatives **5** and especially **6**, their π→π^*^ transition is very close or directly overlapped with the n→π^*^ one, due to an increase in the π orbital energy and a decrease in the energy of the π^*^ orbital. Subsequently, a clear decrease of the relaxation time from 70 h (compound **4**) to 6 h or 24 min for azoderivatives **5** and **6**, respectively, is detected (Figure 7).

**Figure 8:**
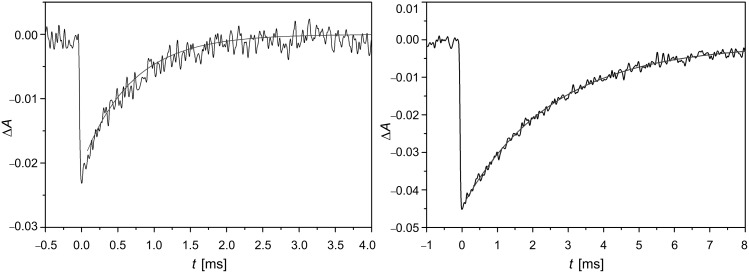
Transient absorption generated by UV irradiation (λ = 355 nm) for azo-dyes **8** (right) and **9** (left) in ethanol at 298 K ([**AZO**] = 20 μM, λ_obs_ = 420 nm for **8** and 405 nm for **9**). From [56] – Reprinted with permission from *Org. Lett.* © 2010, American Chemical Society.

**Figure 9:**
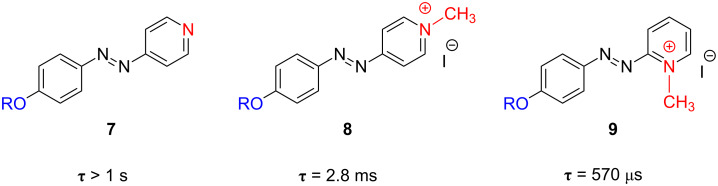
Effect of the presence of a positively charged nitrogen as an electron-withdrawing group on the thermal relaxation time at 298 K, τ, for the *type-I* azoderivatives **8** and **9**.

According to the trend observed for azoderivatives **4**, **5** and **6** (Figure 7), the design of a new series of azoderivatives, bearing a pyridine ring (compound **7**) or a pyridinic cation (compounds **8** and **9**) as an electron-withdrawing group, was carried out. This feature allowed the creation of azo-dyes with a much stronger push–pull configuration than the nitro-substituted compound **6** and, thereafter, faster thermal isomerisation rates were obtained. As a consequence, the *trans*-**8** and *trans*-**9** exhibit intense peaks centred at 410 and 400 nm, respectively, corresponding to the allowed π→π* transition, which is totally overlapped with the n→π* one and highly shifted to the visible zone of the electromagnetic spectrum. It should be highlighted that this band is red-shifted by 50–60 nm with respect to that of the non-push–pull pyridine precursor **7**, which exhibits its π→π* transition at ca. 350 nm, and also 25–35 nm red-shifted from the previously mentioned cyano- or nitro-azobenzene derivatives. This notable red-shift of the absorption wavelength arises from the strong charge transfer from the alkoxy group to the positively-charged nitrogen atom of the pyridinium salt. Figure 8 shows the transient absorption generated upon pulsed laser irradiation of ethanol solutions of azo-dyes **8** and **9** at 298 K. The relaxation times for *cis*-**8** and *cis*-**9** are 2.8 ms and 570 µs, respectively (Figure 9).

**Figure 10:**
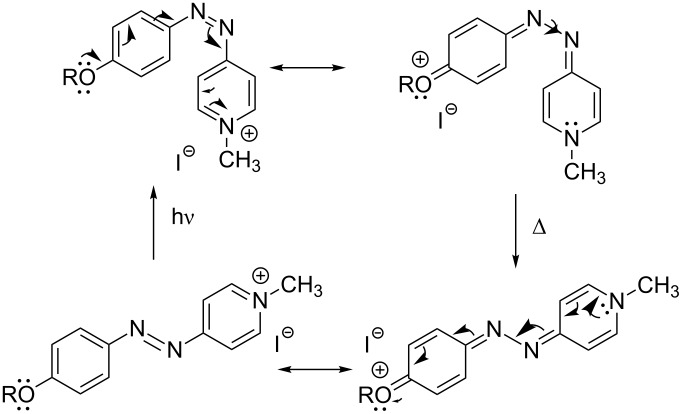
Mechanism proposed for the thermal *cis*-to-*trans* isomerisation process for the push–pull azopyridinium methyl iodide salts **8** and **9**. From [56] – Reprinted with permission from *Org. Lett.* © 2010, American Chemical Society.

Figure 10 depicts the proposed mechanism for the thermally-activated *cis*-to-*trans* isomerisation of the *cis* isomers of the push–pull azopyridinium methyl iodide salts **8** and **9**. The strong electron transfer from the alkoxy group to the pyridinium salt produces a partial breaking of the double N–N bond of the azo moiety, thereby facilitating the rotation around this bond to recover the more stable initial *trans* configuration in a quick fashion [56].

### *Type-II* azoderivatives: photochromic switches acting within the milliseconds time scale, based on chromophores able to establish azo-hydrazone tautomeric equilibria

Hydroxy-substituted azobenzenes are a very interesting family of rapidly thermally isomerising azoderivatives, which have been applied successfully not only for light-driven optical switching applications [43], but also as photoactive monomers in elastomers for light-sensitive artificial muscle-like actuators [57]. In 2005, Kojima et al. found that 4-hydroxyazobenzene (**11**), the simplest azophenol, was endowed with a fast thermal *cis*-to-*trans* isomerisation process [58]. In fact, further experiments with this compound demonstrated a relaxation time of 205 ms for its back reaction at room temperature in ethanol [59].

**Figure 11:**
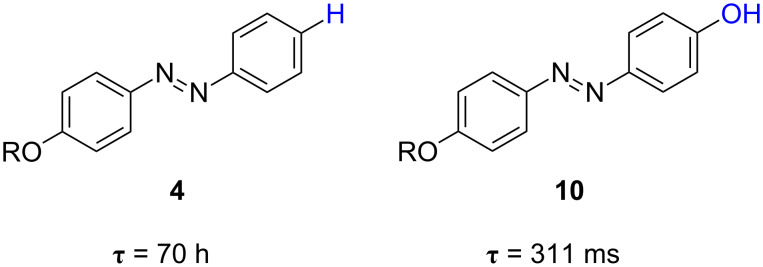
Comparison between the thermal relaxation time at 298 K, τ, for the azoderivative **4** (*type-I*) and the azophenol **10** (*type-II*).

The comparison between the relaxation time for the thermal *cis*-to-*trans* isomerisation of 4-(5-hexenyloxy)azobenzene (**4**) and that of 4-hydroxy-4’-(5-hexenyloxy)azobenzene (**10**) revealed very different behaviours. While the azoderivative **4** exhibits a very slow thermal isomerisation, which can be nicely followed by conventional time-resolved absorption spectroscopy, the thermal back reaction of azophenol **10** must be determined by the laser flash-photolysis technique. Indeed, the thermal *cis*-to-*trans* isomerisation kinetics of *cis*-**10** is up to 8 × 10^5^ times faster than that for the nonsubstituted counterpart **4** (311 ms versus 70 h, respectively, Figure 11). This feature provides evidence for a clear influence of the phenol group on the thermal isomerisation process. The same conclusion can be extracted when comparing the isomerisation kinetics of azophenol **11** with that of azobenzene.

**Figure 12:**
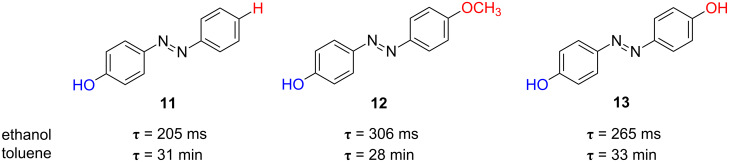
Solvent effect on the thermal relaxation time at 298 K, τ, for the *type-II* azophenols **11**–**13**.

**Figure 13:**
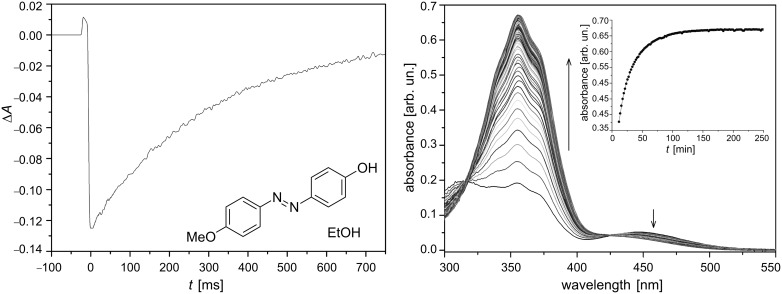
Transient generated by irradiation with UV-light (λ = 355 nm) for the *type-II* azophenol **12** in ethanol (left) and changes in the electronic spectrum of a **12*** cis*-to-*trans* isomerising toluene solution at 298 K (Δ*t* = 120 s, [**12**] = 20 μM). From [59] – Reproduced by permission of the PCCP Owner Societies.

Some insights into the relation between both the chemical structure and the observed kinetic behaviour can be obtained from the study of different *para*-, *ortho*- and *poly*-hydroxy-substituted azobenzenes. There is not only a clear influence of the number and the placement of the hydroxy groups within the azobenzene core, but also of the nature of the solvent used. Both *para-*mono-substituted azophenols **11** and **12** exhibit fast thermal isomerisation kinetics in ethanol at 298 K with relaxation times of 205 and 306 ms, respectively (Figure 12). Remarkably, the relaxation times for these two azocompounds increase in toluene by four orders of magnitude up to 31 and 28 minutes, respectively (Figure 12 and Figure 13). In addition, the analogous *para-*di-substituted azophenol **13** shows a similar behaviour: relaxation times of 33 min and 306 ms in toluene and ethanol, respectively [59].

**Figure 14:**
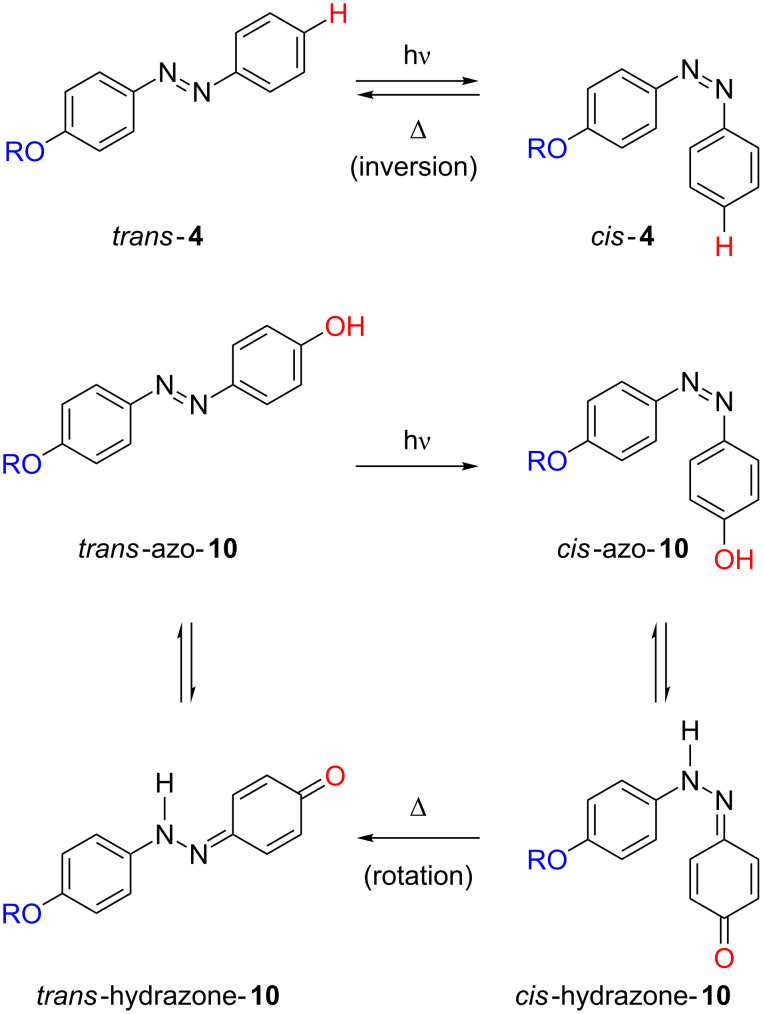
Proposed isomerisation mechanisms for the thermal *cis*-to-*trans* isomerisation of the alkoxy-substituted azoderivative **4** (*type-I*) and the azophenol **10** (*type-II*).

The differential kinetic behaviour detected for azophenols **11**–**13** in toluene and ethanol clearly evidences that the intermolecular interactions established between both the chromophore and the solvent molecules control the rate of the isomerisation process for *type-II* azoderivatives. The formation of intermolecular hydrogen bonds between the nitrogen atom of the azo group and the solvent proton, as well as between the OH group of the azo-dye and the solvent, favours a hydrazone-like electronic distribution with a simple N–N bond, which seems to be the key to the fast thermal isomerisation kinetics observed. In this way, the rotation around the N–N bond facilitates the recovery of the more stable *trans* isomer (Figure 14) [46,59–61].

The completely different kinetic behaviours observed between the alkoxy- and hydroxy-substituted azo-dyes reflect that some alteration in their intimate isomerisation mechanism occurs. The rapid thermal *cis*-to-*trans* isomerisation of the hydroxy-substituted azo-dyes suggests that their back reaction takes place via the rotational pathway, through a polar transition state with a partial breaking of the N–N double bond; however, in the case of the alkoxy-substituted azoderivatives the isomerisation process proceeds by the inversional mechanism (Figure 14).

**Figure 15:**
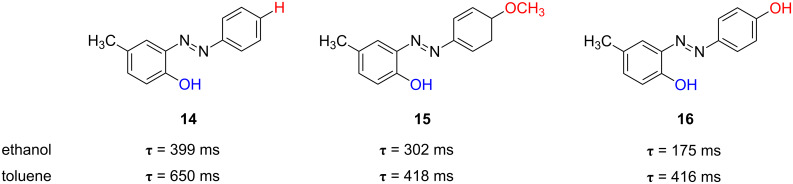
Solvent effect on the thermal relaxation time at 298 K, ***τ***, for the *type-II ortho*-substituted azophenols **14**–**16**.

*Ortho*-substituted azophenols show much shorter *cis*-relaxation times than their *para*-substituted counterparts in toluene solution, e.g., 31 min for *cis*-**11** versus 650 ms for *cis*-**14** (Figure 15). This can be understood bearing in mind that azocompound **11** should undergo dimerization prior to its isomerisation in toluene. Nevertheless, the formation of an intramolecular hydrogen bond [62] in the *ortho-*substituted compound **14** can take place independently of the solvent; this promotes the hydrazone-like tautomeric form and, therefore, the fast isomerisation process can occur in both protic (ethanol) and nonprotic (toluene) solvents. All three *ortho*-substituted azophenols (**14**–**16**) show slightly slower kinetics in toluene than in ethanol, which can be assumed to be because of the impossibility of the former solvent to establish hydrogen bonding with the azo-dye molecules.

**Figure 16:**
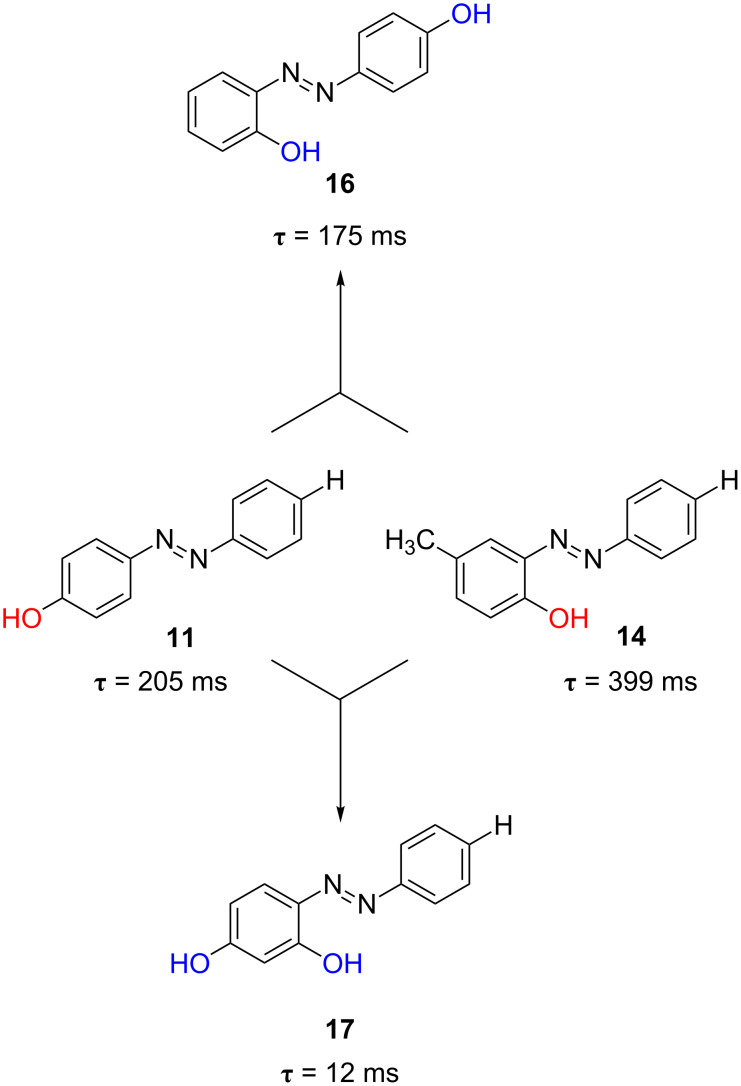
Cooperative effect of the *para*- and *ortho*-hydroxyl groups in azophenol **17**.

In the wake of these promising results, especially of that of the di-substituted azophenol **16** in ethanol, we devised a possible strategy for decreasing the relaxation time of the photochromic switch by placing two hydroxy groups in the same ring of the azobenzene moiety, to obtain an added effect of the two hydroxy groups. The relaxation time of the *ortho*-*para*-di-substituted azophenol *cis*-**17** was 12 ms in ethanol and 53 ms in toluene, respectively. Indeed, the presence of two hydroxy groups in both *ortho*- and *para*-positions of the same ring of the azobenzene core produces a significant acceleration of the process by a cooperative effect. This is not observed when the two hydroxy groups are placed in different rings of the azobenzene molecule (see Figure 16, azophenol **16** versus **17**).

**Figure 17:**
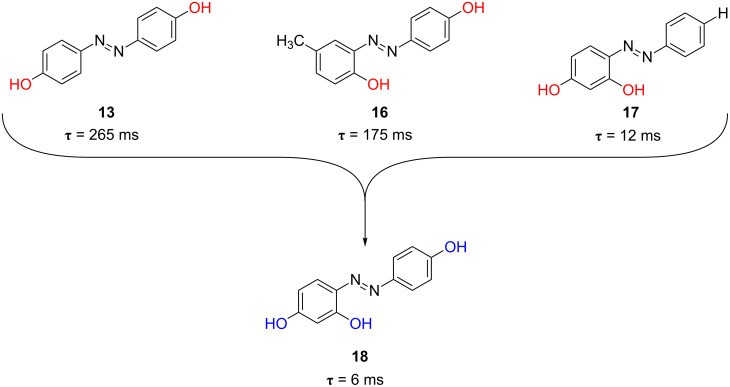
Effect of the *poly*-hydroxylation of the azobenzene core on the thermal relaxation time at 298 K, τ, for the *type-II* azobenzenes: azophenol **18**.

**Figure 18:**
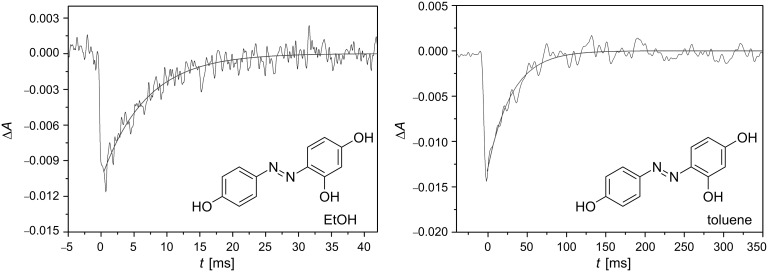
Transients generated by irradiation with UV-light (λ = 355 nm) for the *poly*-substituted azophenol **18** in ethanol (left) and toluene (right) at 298 K. ([**18**] = 20 μM). From [59] – Reproduced by permission of the PCCP Owner Societies.

Moreover, the *poly*-hydroxy-substitution of the azobenzene core, such as in 2,4,4’-trihydroxyazobenzene (**18**), considerably decreases the relaxation time down to 6 and 33 milliseconds in ethanol and toluene, respectively, in comparison with its dihydroxy-substituted counterparts **13**, **16** and **17** (Figure 17). Figure 18 shows the transients for the *poly*-substituted azophenol **18** in both ethanol and toluene at 298 K [59]. In fact, the *cis* isomers of both azoderivatives **17** and **18** show the lowest relaxation times of all the azophenol derivatives presented in this section.

### *Type-III* azoderivatives: optical switches acting within the microsecond time scale, based on azo-dyes exhibiting both azo-hydrazone tautomerism and a push–pull electronic distribution

Among all the azoderivatives presented up to now in this review, azopyridinium methyl iodide salts are the fastest ones (azo-dyes **8** and **9**, see *Type-I* azoderivatives) [56]. These azoderivatives exhibit a strong push–pull configuration, which favours the rotational isomerisation mechanism. The relaxation time of *type-I* azoderivatives ranged from several days to hundreds of microseconds. Specifically, azoderivatives **8** and **9** showed relaxation times of 2.8 ms and 570 μs in alcoholic solutions at 298 K, respectively (see Figure 8). As mentioned above, the relaxation time of *type-II* azophenols ranged from 400 ms to 6 ms in ethanol, depending on the position of the phenol groups within the azobenzene core. However, both types of azoderivatives are still too slow for use as light-driven real-time information-transmitting systems or photo-driven optical oscillators.

The combination in the same molecule of both types of rotational-isomerising azo-dyes was suggested to afford very rapidly thermally isomerising azoderivatives [63]. This section collects different families of azoderivatives that combine the two strategies aforementioned: the presence of powerful electron-withdrawing groups, like nitro or pyridinium salts, and the existence of a phenol function in the convenient positions (2’ and 4’) of the azobenzene core. This feature allows one to obtain highly conjugated systems that extremely favour the thermal isomerisation through the rotational pathway and recovery of the thermodynamically stable *trans* form in a very quick fashion. In this manner, the thermal *cis*-to-*trans* relaxation time for these azoderivatives decreases considerably and, therefore, a further increase in the information transmission capability of the final photochromic switch is observed.

**Figure 19:**
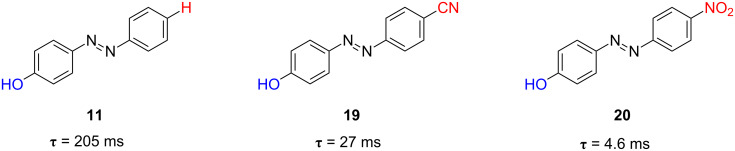
Effect of the introduction of electron-withdrawing groups in the position 4’ of the azophenol structure on the thermal relaxation time at 298 K in ethanol, τ, for the azoderivatives **11** (*type-II*) and **19** and **20** (*type-III*).

**Figure 20:**
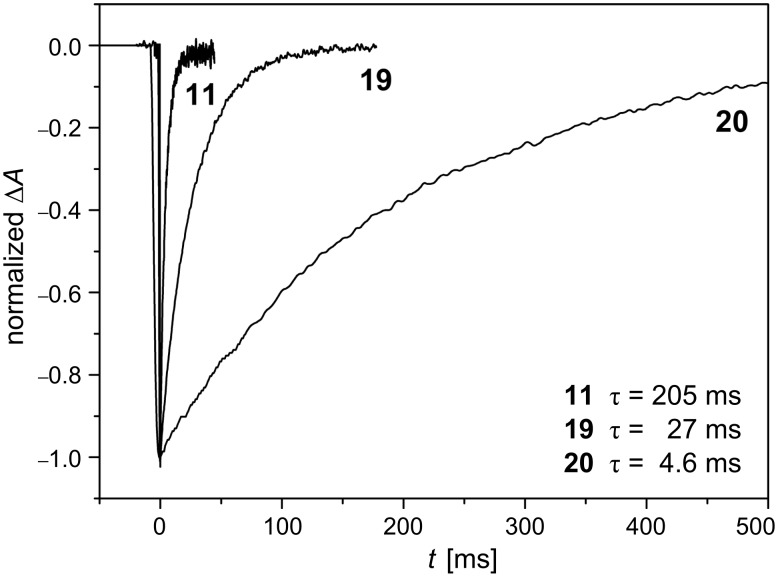
Transient absorptions generated by UV irradiation (λ = 355 nm) of azo-dyes **11** (*type-II*), **19** (*type-III*) and **20** (*type-III*) in ethanol at 298 K ([**AZO**] = 20 µM, λ_obs_ = 370 nm). From [63] – Reproduced by permission of the Royal Society of Chemistry.

In order to get an idea of the benefits of this strategy it is worth comparing the first the relaxation times of parent azocompounds. Azophenol **11** shows a relaxation time of 205 ms for its thermal isomerisation in ethanol at 298 K (see Figure 12). The introduction of the electron-withdrawing cyano group in the position 4’ of the azophenol (compound **19**) caused the relaxation time of the 4’-cyanoazophenol **19** to decrease 10-fold compared to that of the parent azophenol **11** (27 ms versus 205 ms). The relaxation time decreased even further for the 4’-nitroazophenol **20** showing a τ value of 4.6 ms (Figure 19 and Figure 20). Hence, upon going from the azophenol **11** to its push–pull nitro counterpart **20**, the thermal *cis*-to-*trans* isomerisation process becomes faster and, consequently, an increase of the information transmission capability of the photochromic switch is observed.

**Figure 21:**
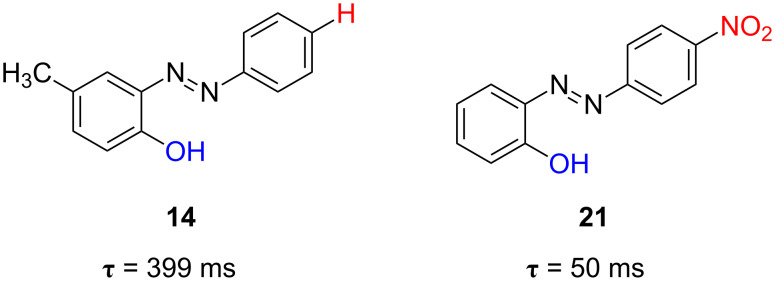
Effect of the introduction of the hydroxyl group in the position 2’ of the push–pull azo-dye on the thermal relaxation time at 298 K in ethanol, τ, for azoderivatives **14** (*type-II*) and **21** (*type-III*).

The placement of the hydroxy group in the position 2 has been also studied for the *type-III* azophenols. In contrast to that found for the *type-II* azophenols, a notably increase of the relaxation time was detected for the *ortho*-substituted azophenol containing the electron-withdrawing nitro group **21** (τ = 50 ms, Figure 21) with respect to the corresponding non-push–pull counterpart (τ = 399 ms) in ethanol at 298 K. These values are substantially larger than those of the corresponding *para*-hydroxy-substituted azo-dyes **11** (205 ms) and **20** (4.6 ms), respectively (Figure 19).

**Figure 22:**
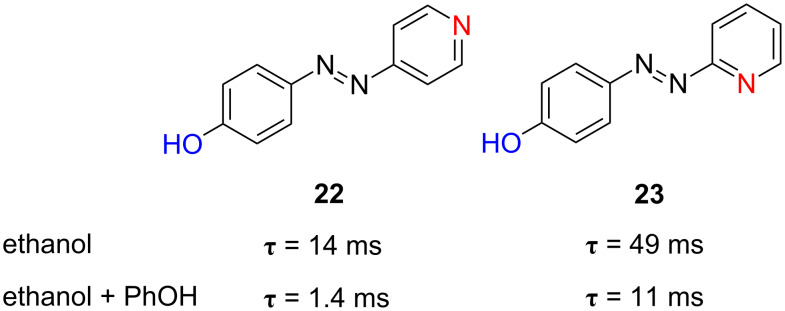
Effect of the substitution of a benzene ring by a pyridine one on the thermal relaxation time in ethanol and effect of 1 equiv phenol added at 298 K, τ, for *type-III* azoderivatives **22** and **23**.

The substitution of one of the benzene rings of the azo-dye by a pyridine one, a π-electron-deficient heterocycle, has been also considered. In this case, the hydrogen bonding established between ethanol and the pyridine moiety places a positive-charge density over the pyridine nitrogen, which increases susbsequently the push–pull electronic distribution of the azo-dye (compounds **22** and **23**). As observed in Figure 22, the relaxation time of these two azocompounds in ethanol is 49 ms and 14 ms, respectively, substantially shorter than that of the parent 4-hydroxyazobenzene **11** (205 ms), but too large compared to that of the *para*-nitro-substituted azo-dye **20** (4.6 ms).

Additionally, when phenol is added to the ethanol solution, the thermal *cis*-to-*trans* isomerisation kinetics is accelerated. Phenol forms a stable hydrogen bond with the nitrogen atom of pyridine. Indeed, addition of 1 equiv phenol increases the isomerisation rate of azo-dyes **22** and **23** by a factor of 10 and 4.5, respectively (Figure 22). The differential effect can be associated with the major difficulty of the pyridinic nitrogen to establish a hydrogen bond with phenol in the position 2 of the azobenzene core due to steric hindrance.

**Figure 23:**
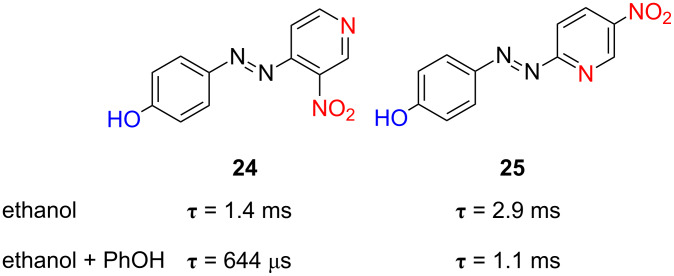
Influence of the introduction of additional electron-withdrawing nitro groups in the pyridine ring on the thermal relaxation time in ethanol and effect of 1 equiv phenol added at 298 K, τ, for *type-III* azoderivatives **24** and **25**.

The introduction of additional nitro groups in the *ortho*- and *para*-positions with respect to the azo function has been also considered (compounds **24** and **25**). These two azoderivatives show relaxation times of 2.9 ms and 1.3 ms in ethanol, respectively, which decrease further down to 1.1 ms and 644 µs in the presence of phenol (Figure 23). This demonstrates that the generation of push–pull systems by the establishment of hydrogen bonds between phenol groups and the pyridinic nitrogen, with the subsequent generation of a deficient electron density in the latter, is a versatile option towards the generation of fast information-transmitting photochromic switches.

**Figure 24:**
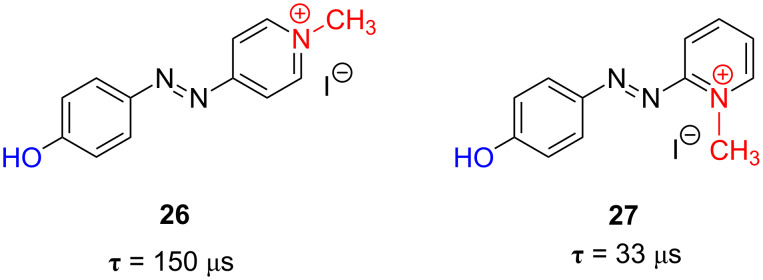
Chemical structure and thermal relaxation time in ethanol at 298 K, τ, for the *type-III* azoderivatives **26** and **27**.

Extending the concept even further, azopyridines with a permanent positively charged nitrogen should increase the kinetics and the stability of the final photodriven oscillator. Methylation of the pyridine nitrogen of azo-dyes **22** and **23** was carried out to afford the corresponding pyridinium methyl iodide salts **26** and **27**. Thus, the methyl hydroxy-substituted azopyridinium salts **26** and **27** present relaxation times of only 150 µs and 33 µs, respectively, at room temperature in ethanol (Figure 24). To the best of our knowledge, these are the fastest thermally isomerising azophenol derivatives heretofore reported in the literature [63].

**Figure 25:**
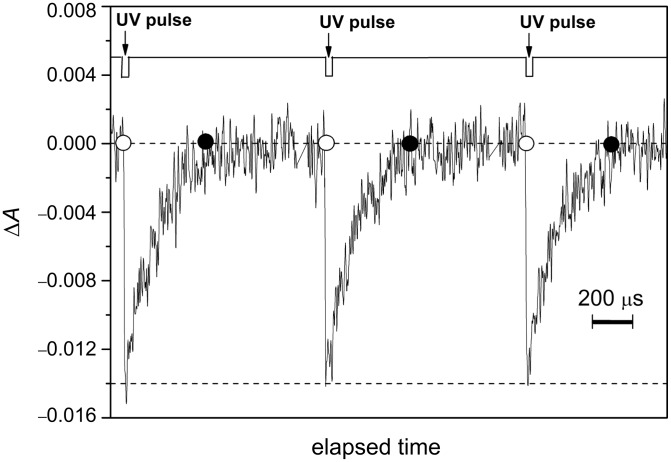
Oscillation of the optical density of an ethanol solution of azo-dye **26** generated by UV-light irradiation (λ = 355 nm, 5 ns pulse width) at 298 K ([**26**] = 20 μM, λ_obs_ = 420 nm). From [63] – Reproduced by permission of the Royal Society of Chemistry.

Because of their very fast thermal isomerisation rate, **26** and **27** are the best candidates to be applied as fast information-transmitting photochromic switches. Figure 25 shows the information-transmission capability of azo-dye **26** with time. The repeatability of the different optical oscillators was tested by submitting them to consecutive UV-irradiation–dark cycles. The optical photochromic switches reported show no fatigue (Figure 25).

## Conclusion

This review compiles very recently reported advances with regard to rapidly thermally isomerising azoderivatives, which are valuable candidates to be applied as fast information-transmitting photochromic switches. Two structural features should be present in the azobenzene molecule for this purpose: a push–pull electronic distribution together with the existence of a hydroxy group that allows the keto–enol equilibrium to be established. These two factors allow the thermal back isomerisation process of the azo-dye to proceed through the rotational pathway, and, consequently, the thermodynamically stable *trans* form of the azo-dye can be restored in a very quick fashion. Indeed, those azoderivatives that combine both structural factors exhibit an information-transmitting capability within tens of microseconds.
